# Exploring the potential of taurolidine in inducing mobilization and detachment of colon cancer cells: a preliminary in-vitro study

**DOI:** 10.1186/s40360-022-00572-8

**Published:** 2022-06-13

**Authors:** Agata Mikolajczyk, Veria Khosrawipour, Hien Lau, Shiri Li, Pawel Migdal, Maya Karine Labbé, Wojciech Kielan, Jakub Nicpon, Sven Stieglitz, Tanja Khosrawipour

**Affiliations:** 1 Department of Biochemistry and Molecular Biology, Wroclaw University of Environmental and Life Sciences, 50-375 Wroclaw, Wroclaw, Poland; 2 2nd Department of General Surgery and Surgical Oncology, Wroclaw Medical University, 50-556 Wroclaw, Wroclaw, Poland; 3 Department of Surgery, University of California Irvine (UCI), Orange, CA 92868 USA; 4 Department of Surgery, Weill Cornell Medical College New York Presbyterian Hospital, New York, 10065 USA; 5 Department of Environment, Hygiene and Animal Welfare, Wroclaw University of Environmental and Life Sciences, 50-375 Wroclaw, Wroclaw, Poland; 6 School of Dentistry, Wroclaw Medical University, 50-367 Wroclaw, Poland; 7 Department of Surgery, Faculty of Veterinary Sciences, Wroclaw University of Environmental and Life Sciences, 50-375 Wroclaw, Wroclaw, Poland; 8 Department Pulmonary Medicine, Petrus-Hospital Wuppertal, University of Witten-Herdecke, 42283 Wuppertal, Wuppertal, Germany; 9 Department of Surgery (A), University-Hospital Düsseldorf, Moorenstrasse 5, 40225 Düsseldorf, Düsseldorf, Germany; 10 Medical faculty, Heinrich-Heine University Düsseldorf, Düsseldorf, Germany

**Keywords:** Taurolidine, Cancer, Chemotherapy, Cytoreductive surgery, Metastasis, Cytotoxicity

## Abstract

**Background:**

Recently, taurolidine has been intensively studied on a variety of in-vitro cancer cell-lines and first data exhibit encouraging antitumoral effects. While the clinical use of taurolidine is considered, some studies with in-vivo experiments contradict this beneficial effect and even indicate advanced cancer growth. The aim of this study is to further investigate this paradox in-vivo effect by taurolidine and closely analyze the interaction of cancer cells with the surrounding environment following taurolidine exposure.

**Methods:**

HT-29 (ATCC® HTB-38™) cells were treated with taurolidine at different concentrations and oxaliplatin using an in-vitro model. Morphological changes with respect to increasing taurolidine dosage were visualized and monitored using electron microscopy. Cytotoxicity of the agents as well as extent of cellular detachment by mechanical stress was measured for each substance using a colorimetric MTS assay.

**Results:**

Both taurolidine and oxaliplatin exhibit cell toxicity on colon cancer cells. Taurolidine reshapes colon cancer cells from round into spheric cells and further induces cluster formation. When exposed to mechanical stress, taurolidine significantly enhances detachment of adherent colon carcinoma cells compared to the control (*p* < *0.05*) and the oxaliplatin group (*p* < *0.05*). This effect is dose dependent.

**Conclusions:**

Beside its cytotoxic effects, taurolidine could also change mechanical interactions of cancer cells with their environment. Local cancer cell conglomerates could be mechanically mobilized and may cause metastatic growth further downstream. The significance of changes in cellular morphology caused by taurolidine as well as its interaction with the microenvironment must be further addressed in clinical cancer therapies. Further clinical studies are needed to evaluate both the safety and efficacy of taurolidine for the treatment of peritoneal surface malignancies.

## Background

**Fig. 1:**
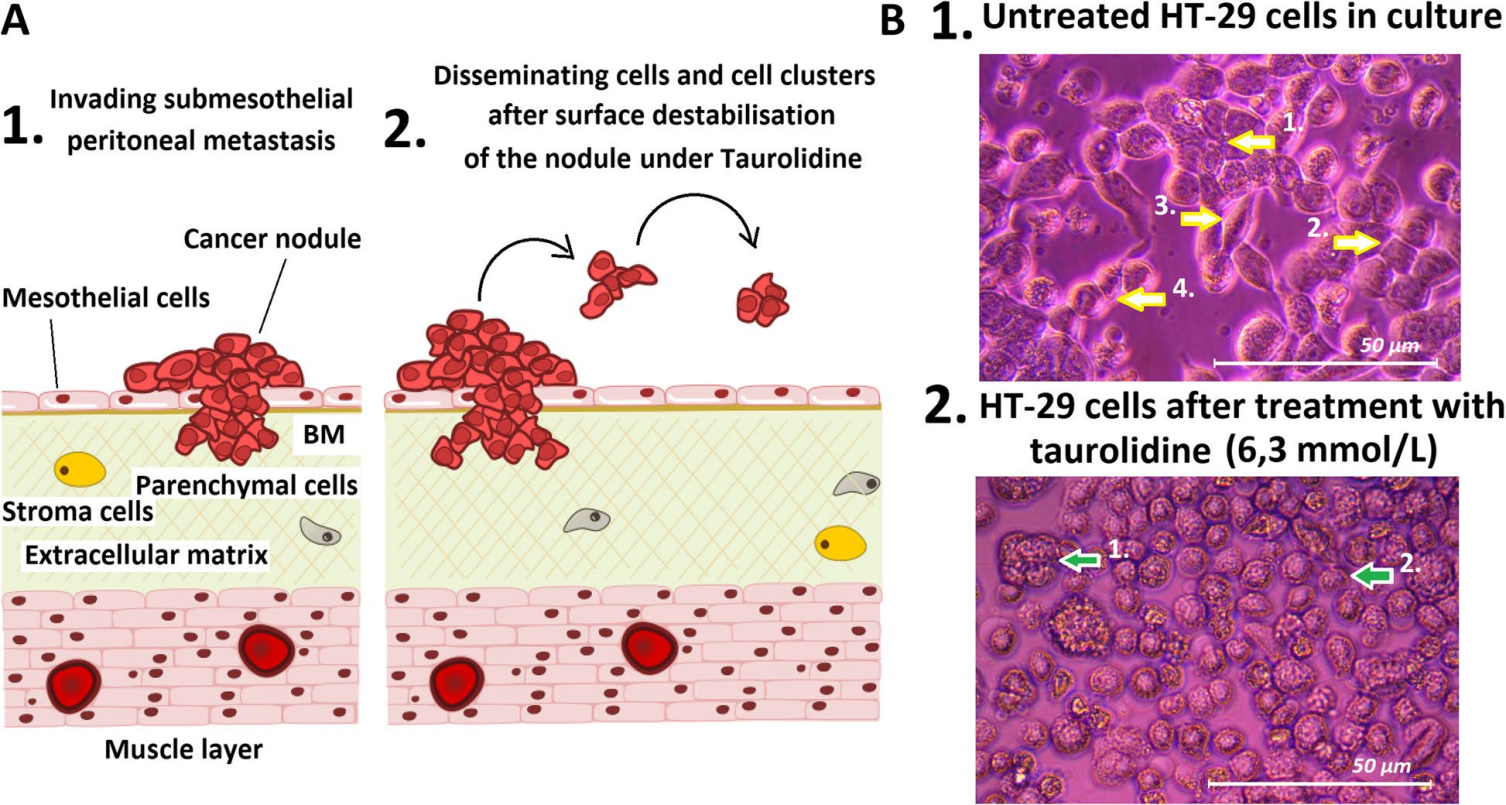
**A** Model of surface metastasis in PM and its potential further enhancement by taurolidine despite its antitumoral effects on in-vivo colon cancer cells. 1) Model of peritoneal metastatic nodule and 2) mobilization of cells and cell-clusters by destabilization of the cancer nodule by taurolidine. **B** Light-microscopy of in-vitro colon cancer (HT-29) cell cultures (Magnification 40X). 24 h after treatment structural changes are visible in the taurolidine group. 1). Untreated cells with their typical features (Yellow arrows: 1. compact/tight interaction 2. Polyhexagonal wall formation 3. Polystructural shape with interaction to the bottom 4. Cells are within a narrow visual band and therefore better visible 1) and 2.) Cells treated with taurolidine (green arrows: 1. floating round shape polycell clusters and 2. single detached shere shape cells with little contact to the bottom and the surrounding cells)

In recent years, the role of extracellular interactions between cancer cells and their microenvironment have been the focus of numerous studies. In fact, local cancer growth, tissue invasion and tumor metastasis are influenced by many extracellular factors [1, 2]. Some of these key factors include local cancer cell migration [3–5]. While some factors influencing, enhancing or reducing cancer cell migration have already been identified [5–10], many more are currently under evaluation. Recently, some medications which influence and inhibit interaction of cancer cells have been evaluated and identified for possible clinical applications [11]. While of major importance, the effects on extracellular interaction remain unknown for many of these substances. Taurolidine has been identified as a cytotoxic agent and its effectivity has been documented for various cancer-cell lines [12–14]. In clinical applications, taurolidine has been mostly used due to its antibacterial qualities. These applications include peritoneal lavage in children or in patients receiving peritoneal dialyses and display signs of peritonitis [15, 16]. In some cases, taurolidine is also used to block central venous catheters [17–19]. While in-vitro and animal data mostly support taurolidine's tumoricidal abilities [20, 21], there are a few published clinical studies which evaluate the role of taurolidine as a chemotherapeutic agent [22]. Current scientific literature lacks a gold-standard level 1 randomized clinical trial to evaluate taurolidine's potential antineoplastic benefits. Further clinical trials are currently in process. Additional studies are required to further clarify the role of taurolidine in modern cancer treatments. In fact, first in-vivo studies have presented two major problems following taurolidine application, including some cases of hepatotoxicity following intraperitoneal delivery of taurolidine [12, 23]. This issue seems manageable and does not limit the potential for clinical applications. The second and more serious concern is that some in-vivo studies indicate increased metastatic tumor growth following taurolidine application [24, 25]. Moreover, there are some indications that taurolidine might even cause structural changes in the cellular morphology of exposed cells [26]. This discrepancy between promising in-vitro and concerning in-vivo results has yet to be investigated. This study is the first to thoroughly investigate the behavior of taurolidine on colon cancer cells and assess its potential as well as its risks for therapeutic applications. More specifically, we aim to investigate its potential in enhancing metastatic growth due to its cell detachment effects. The dissemination of cancer cells within the peritoneum has been considered the main mechanism of peritoneal metastasis (PM) (Fig. 1 A).

## Methods

### Cell culture

Human colorectal cancer cell line HT-29 (ATCC® HTB-38™) was obtained from the Institute of Immunology and Experimental Therapy (Wrocław, Poland). HT-29 cells were grown in Dulbecco’s modified Eagle’s medium (DMEM—high glucose, Sigma-Aldrich; Merck KGaA, Darmstadt, Germany) supplemented with 10% heat-inactivated fetal bovine serum (FBS, Gibco, Thermo Fisher Scientific, Poland), 2 mmol/L glutamine, 100 IU/mL penicillin, and 100 μg/mLstreptomycin (all purchased from Sigma-Aldrich; Merck KGaA) and cultured at 36 °C in a humidified 5% CO_2_/air atmosphere. Cells were seeded in 24-well plates (TC Plate 24 Well, Standard, F, Sarstedt AG &Co. KG, Germany) at a density of 1.4 × 10^5^ per well and incubated under standard conditions.

### Drug dosage

In this study we used different taurolidine concentrations (Taurolin® Ringer, Berlin-Chemie AG, Germany), which were dissolved in medium. The concentrations were 0.045%, 0.06%, 0.09%, 0.135% and 0.18%, respectively. Oxaliplatin (Medoxa, medac GmbH, Wedel, Germany) was diluted in 5% glucose solution [27, 28]. The concentration in each well was 0.24 mg/ml (= 0,6 mmol/L) which corresponds to common HIPEC application amounts of 960 mg/4 L (480 mg/m^2^). Each well received 30 µl of the concentrated Oxaliplatin solution (184 mg/150 ml). Untreated cell cultures were used as control.

### Exposure time

Following a growth period of 48 h, culture medium of HT-29 cells was removed and replaced with 150 µL of fresh medium. Then, cells were treated with oxaliplatin and taurolidine at various concentrations for one hour. After one hour of exposure, medium was removed, and fresh medium was added. Cells were incubated for another 24 h. Then, wells were rotated at 300 rpm for 2 min. Following the exposure period, the entire solution was aspirated from the cells and transferred into separate wells. Empty wells were filled with fresh medium. Cell incubation continued for another 24 h at 36 °C and 5% CO_2_ before MTS proliferation assay was performed.

### MTS test

The cell proliferation assay (CellTiter 96®AQ_ueous_ One Solution assay, Promega, Poland) was performed according to the manufacturer’s instruction with modifications. Briefly, the medium was removed from each well and replaced with 0.3 mL of fresh DMEM. Next, an MTS-based reagent was added to each well and absorbance was measured at 490 nm following 1 h of incubation at 36 °C and 5% CO_2_ using a microplate reader (Tecan, Basel, Switzerland).

### Trypan Blue Exclusion Assay

To evaluate cell population density, a Trypan Blue (TB) dye exclusion assay with CellDrop automated cell counter (De Novix, Wilmington, USA) was used. After performing the procedure of rotating and transferring cells, HT-29 cells were grown in initial wells for 24 h and transferred into wells for 48 h. Cells were trypsynized in each well and neutralized with a total volume (100 µl) of final suspension. 10 µl of cell suspension were taken, mixed with 10 µl of TB and measured by CellDrop automated cell counter using trypan blue counting mode. Number of viable and dead cells per milliliter was measured.

### Analyses via light microscopy (LM)

After 24 h after exposure to taurolidine (6.3 mmol/L), HT-29 cells were visualized using a light microscope (Primovert, Zeiss, Göttingen, Germany) with the support of the FX-5000 version 1.0 program, installed on a connected computer. In an in-vitro setting, cells treated with taurolidine were compared to untreated cells.

## Analyses via electron microscopy (EM)

Four cell groups were further analyzed via cryogenic scanning electron microscopy (cryo-SEM) analysis. For that purpose, cells were cultured on round glass slides (cover slides). Cells received either low taurolidine exposure at 1.575 mmol/L or high exposure at 6.3 mmol/L. Alternatively, they received oxaliplatin exposure at 0.6 mmol/L according to the described protocol. Slides were then removed and subjected to cryo-SEM analysis. The fourth group (control) were untreated cells. The probe was washed with Dulbecco’s phosphate buffered saline (DPBS, Sigma-Aldrich) and fixated in 2.5% glutaraldehyde solution (Sigma-Aldrich). After fixation, samples were washed in PBS, rinsed in ultrapure (sterilized through 0.1 µm filter) deionized water, mounted on cryo-shuttle using OT/colloid graphite mixture and plunged in liquid nitrogen. Frozen specimens were quickly transferred to a cryo-preparation chamber (Cryo Quorum PP3010T) and sputtered with a conductive layer of platinum at -140 °C. Then, specimen were transferred to the microscopy chamber while maintaining the same temperature of -140 °C (Auriga60, Zeiss). Sample were analyzed using 2 kV of acceleration voltage using In Lens and SE2 secondary electron detectors.

### Statistical analysis

**Fig. 2:**
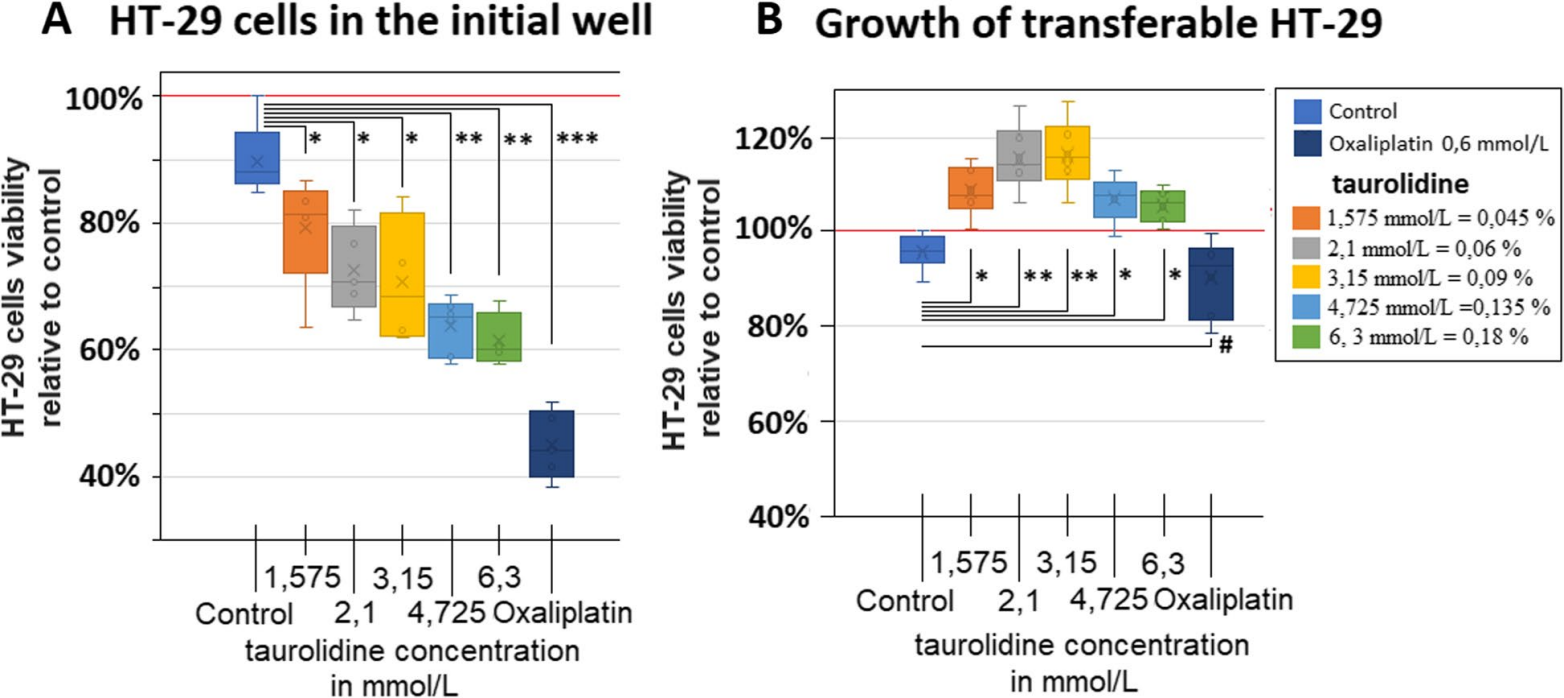
**A** Viability of HT-29 cells following exposure to taurolidine and oxaliplatin (OX) and exposure to mechanical stress (rotation). **B** Growth of floating HT-29 cells following mechanical stress (rotation) to the initial well and transfer into a separate well

All experiments were independently performed three times. The statistical analyses were performed using Sigma Plot 12 (Systat Software Inc., California, USA). Probability *(p)* values were considered as follows: * = *p* < 0.05; ** = *p* < 0.01;; *** = *p* < 0.005; # = *p* > 0.05, with *p*-value < 0.05 considered statistically significant. The measured absorbance levels of the MTS test were compared to controls, where maximum absorbance in the control corresponded to 100% and all further levels were in reference to this value (Fig. 2A and B).

## Results

### The effect of mechanical stress on Taurolidine and Oxaliplatin treated HT-29 cells

**Fig. 3:**
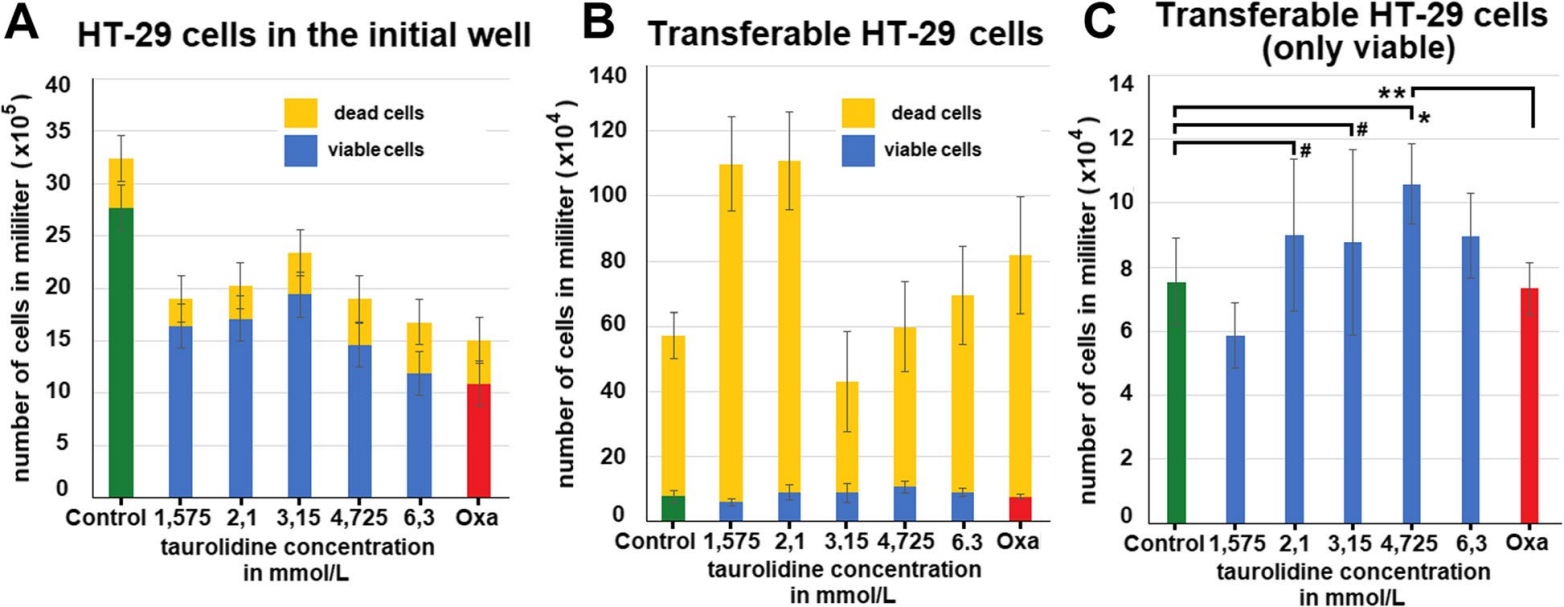
Cell count via trypan blue of HT-29 cells following exposure to taurolidine, oxaliplatin (OX) and mechanical stress (rotation). **A** Number of HT-29 cells remaining in the initial well following rotation. **B** Total number of transferred HT-29 cells following mechanical stress (rotation) and **C**) number of viable cells only

After treatment and exposure to rotational mechanical stress, a higher number of cells were seen floating in the taurolidine treatment groups when using light microscopy. Increased detachment was observed with higher taurolidine dosage. In contrast, only little cell detachment was detected in the control and the oxaliplatin treatment group. The viability of the wells 24 h after treatment shows significantly lower viability following taurolidine treatment (*p *< 0.05 for T = 1.575 mmol/L, T = 2.1 mmol/L, T = 3.15 mmol/L and *p* < 0.01 for 4.725 mmol/L and 6.3 mmol/L) compared to the control group (Fig. 2A). The lowest viability was detected in oxaliplatin group 0,6 mmol/L (*p *< 0.005) when compared to the control group (Fig. 2A). In contrast, we detected in taurolidine treated cells an increase in cell viability of transferred cells from the prior experiment (Fig. 2B). Measured viability of taurolidine groups was significantly higher (T = 1.575 mmol/L, 4.725 mmol/L and 6.3 mmol/L), (*p* < 0.05) and T = 3.15 mmol/L, T = 2.1 mmol/L (*p* < 0.01) than in control groups. Viability appears to peak at a concentration of around T = 2.1 mmol/L and T = 3.15 mmol/L. While the oxaliplatin group 0.6 mmol/L seems to display lower viability numbers than the control, this observation is not statistically significant (*p* > 0.05). The measurement of dead and viable cells using trypan blue confirmed our observations. The amount of viable HT-29 cells in the initial well was highest in the control group and lower in the other groups (Fig. 3A). In the transferred well, an overall high number of dead cells vs. viable cells was detected in all groups (Fig. 3B). In the transferred well, the highest number of viable cells was noted in the wells with T = 2.1 mmol/L, T = 3.15 mmol/L, 4.725 mmol/L and 6.3 mmol/L. However, this effect was only significantly higher for T = 4.725 mmol/L vs. control (*p* < 0.05) and vs. Oxaliplatin (*p* < 0.01) (Fig. 3C).

## Observable visual differences under light microscopy and changes induced by taurolidine

Images of cells exposed to taurolidine as well as control cells are presented in Fig. 1B. We observed single, spheric formed cells as well as single, detached cells floating in the medium following treatment with taurolidine. Moreover, cell clusters ranging from two to up to approximately 50 cells were observed. Neither controls nor oxaliplatin treated cancer cells showed similar behavior.

## Confirmation of changes and further visual details under electron microscopy (EM)

**Fig. 4:**
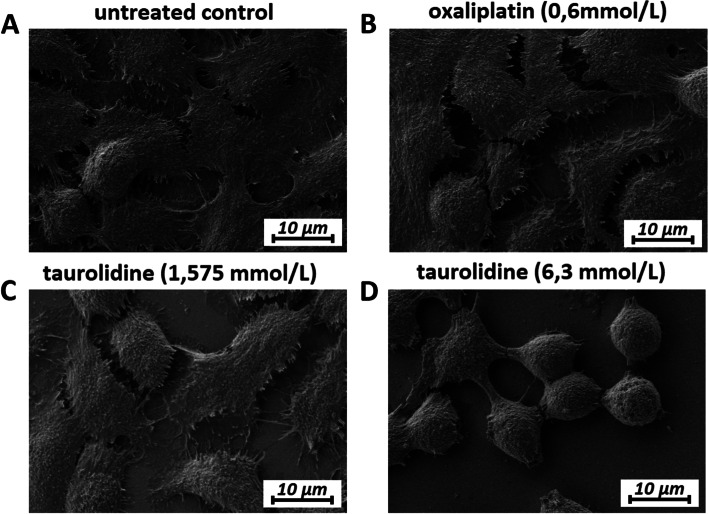
Scanning-electron microscopy (SEM) of in-vitro colon cancer (HT-29) cell cultures. Structural changes on the extracellular matrix and cell–cell interaction are visible in the taurolidine group 24 h after treatment. Moreover, sphere formation can be observed in the high-dose taurolidine group. Untreated control (**A**), Oxaliplatin (**B**) low-dose Taurolidine (**C**) and high-dose Taurolidine (**D**)

**Fig. 5:**
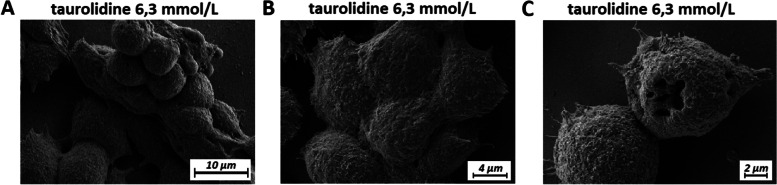
Scanning-electron microscopy (SEM) of colon cancer (HT-29) cell cultures 24 h after taurolidine treatment (6,3 mmol/L). Conglomeration of cancer cells to larger units and cluster (**A** and **B**) and beginning detachment of cell cluster from the surface **A** “Punch-like” lesions of cancer cells following high-dose taurolidine exposure **c**

Cryo-SEM was used to study cell morphology following taurolidine and oxaliplatin treatment. Structural analyses of the extracellular cell surface revealed a similar texture between controls, cells treated with oxaliplatin, and cells treated with low dose taurolidine. However, with increasing taurolidine dose the morphology of the cells changed. Taurolidine might destabilize the intercellular cohesion of cancer cells by reducing the amount of extracellular and adhesive matrix in-between cells (Fig. 4). In fact, taurolidine reshaped colon cancer cells and induced spheric cell formation in individual cells as well as conglomerate cell clusters (Figs. 4 and 5 A, B). This cluster formation was neither observed in control groups nor in the oxaliplatin group. In some cases, taurolidine can also cause further destabilization of the cell wall, including “punch-like” defects of the outer wall (Fig. 5C).

## Discussion

The concept of disseminating cancer cells and cell clusters on the peritoneal surface is considered the main mechanism behind PM (Fig. 1A). While there is reliable data supporting the cytotoxic properties of taurolidine, we were able to observe how taurolidine also alters colon cancer cell morphology. It is possible that initially adherent cells start to mobilize and reorganize into floating cell clusters under the influence of taurolidine. These cell clusters, which still contain viable cells, have much less contact with the bottom of the well as observed in-vitro. Slightly adherent cells could resettle much easier if they are subjected to a fluid stream or any other mechanical replacement. To our knowledge, this study is the first to describe this phenomenon for taurolidine. In fact, this could potentially explain why the use of taurolidine has been associated with a higher degree of metastasis [24] despite its antitumoral properties. By means of taurolidine, viable cell-clusters adherent to the vascular endothelial could be mobilized, washed away and travel further downstream where they could deposit and grow as new metastasis. A similar effect has partly already been observed in local treatment of surface malignancies [25]. The phenomenon of mechanical mobilization of cell-clusters via chemical solutions has recently been discussed. The first attempt to potentially use this effect has been suggested for Ethylenediaminetetraacetic acid (EDTA) in the treatment of PM [11]. EDTA has been long known to mobilize cell clusters from the underlying surface. This effect may be of potential use in the chemical mobilization of adherent malignant cells from the surface which could then be washed out to achieve a cancer free surface. This effect would extend beyond currently available possibilities obtained by cytoreductive surgery (CRS) and hyperthermic intraperitoneal chemotherapy (HIPEC). However, our data suggests that if mobilized cancer cells are not removed, they could result in new, distant metastatic growth. Our data further indicate that while taurolidine is cytotoxic for cancer cells, it could potentially enhance cancer cell mobility and thus, still increase metastatic growth. The exact study design to investigate taurolidine in a clinical setting should be carefully considered as to prevent potential cancer growth which could result in worse overall outcome. It would be conceivable to combine taurolidine with HIPEC if removal of applied taurolidine/chemolavage can be ensured. The possibility of a curative approach by means of CRS and HIPEC largely depends on complete macroscopic tumor resection followed by a chemotherapeutic lavage. However, residual microscopic tumor cells remaining in the peritoneum can cause PM recurrence after initial remission due to limited drug interaction within tumor cell clusters [29, 30]. Despite warranted criticism of high morbidity and mortality rates following CRS and HIPEC, these procedures promise a potentially curative approach when other therapeutic options fail to do so. This is an important consideration as tumor cell clusters and nodules can be easily overlooked, especially if they are too small for macroscopic detection, thus resulting in recurrent PM. Detection of tumor nodules < 1 mm is unlikely if no additional visual devices are used. However, enhanced cancer cell mobilization should be considered in any clinical setting and application, and thus be counteracted. This present study is indicative of possible dangers of an uncalculated taurolidine use.

### Limitations

This in-vitro study offers preliminary data. No certain conclusions can be drawn to explain the observed morphological changes and their underlying biological mechanisms on a cellular or even biomolecular level. Taurolidine related detachment and seeding of cancer cells must be further investigated and proven in the in-vivo setting. Evaluating the adhesive-related molecules through gene or protein expression analysis would be required to further confirm the effect of taurolidine on cell detachment. Also, evaluating genes involved in the process of metastasis should be considered. Finally, further in-vivo studies should be considered to confirm the migration and invasion of treated cancer cells.

## Conclusion

Beyond its mere antitumoral effects, taurolidine causes detectable morphological changes in cancer structure which could be of high clinical relevance for cancer treatment. Therefore, taurolidine could potentially even enhance cancer metastasis in-vivo despite its in-vitro antitumoral effects. Due to the limited extent of this study, further studies, including molecular and clinical trials, are required to assess potential benefits and risks of taurolidine therapy in cancer treatments.

## Data Availability

Data can be obtained from the corresponding author upon reasonable request.

## Abbreviations

CG: Control group
CRS: Cytoreductive surgery
cryo-SEM: Cryogenic scanning electron microscopy
HIPEC: Hyperthermic intra-peritoneal chemotherapy
IPC: Intraperitoneal chemotherapy
MTS: 3-(4,5-Dimethylthiazol-2-yl)-5-(3-carboxymethoxyphenyl)-2-(4-sulfophenyl)-2H tetrazolium
OX: Oxaliplatin
PM: Peritoneal Metastasis
